# Quantitative terbium-161 SPECT/CT imaging: demonstrating the feasibility of image-based dosimetry and highlighting pitfalls

**DOI:** 10.1186/s13550-025-01326-3

**Published:** 2025-10-13

**Authors:** Frida Westerbergh, Nicholas P. van der Meulen, Cristina Müller, Andreas Grings, Philipp Ritt, Peter Bernhardt

**Affiliations:** 1 Department of Medical Radiation Sciences, Institute of Clinical Sciences, Sahlgrenska Academy at University of Gothenburg, Gothenburg, Sweden; 2 Laboratory of Radiochemistry, PSI Center for Nuclear Engineering and Sciences, Villigen Villigen-PSI, Switzerland; 3 Center for Radiopharmaceutical Sciences, PSI Center for Life Sciences, Villigen Villigen-PSI, Switzerland; 4 Department of Chemistry and Applied Biosciences, ETH Zürich, 8093 Zürich, Switzerland; 5 Clinic of Nuclear Medicine, University Hospital Erlangen, Erlangen, Germany; 6 ITM Oncologics GmbH, Lichtenbergstrasse 1, Munich 85748 Garching, Germany; 7 Department of Medical Physics and Biomedical Engineering (MFT), Sahlgrenska University Hospital, Gothenburg, Sweden

## Abstract

**Background:**

Terbium-161 (^161^Tb) is a promising β⁻-emitter for theragnostics. However, its complex photon emission pattern—including intense X-rays and low-yield, high-energy γ-emissions—may complicate image-based quantification.

This study aimed to assess the feasibility of accurate SPECT/CT-based ^161^Tb dosimetry through a series of phantom measurements using a GE Discovery NM/CT 670 Pro system. Three collimators were evaluated: extended low-energy general-purpose (ELEGP), low-energy high-resolution (LEHR), and medium-energy general-purpose (MEGP), using two separate energy windows: around the 75 keV γ-peak (± 10%), and around the 49 keV γ-peak and nearby X-rays (40.7–62.9 keV). A clinical OSEM reconstruction algorithm was employed.

**Results:**

On average, the SPECT calibration factors (CFs) were 2-fold higher with ELEGP compared to MEGP and LEHR, and 3-fold higher at 49 keV compared to 75 keV. For each collimator, derived CFs varied substantially depending on measurement and volume-of-interest geometry—more so at 49 keV, compared to 75 keV.

Measurements of two 3D-printed kidney inserts revealed superior visual image quality with LEHR compared to ELEGP and MEGP. Across all collimators, the 75 keV window provided better spatial resolution and contrast than the 49 keV window.

An anthropomorphic phantom study, including a LungSpine phantom with 8 spherical inserts and 3 different background activity levels, demonstrated a greater quantitative accuracy for MEGP compared to LEHR and ELEGP, with statistical significance for both energy windows (*p* ≤ 0.001). Errors were generally larger at 49 keV compared to 75 keV. For the low-energy collimators, considerable septal penetration (e.g., at 292 and 475 keV) was observed, along with systematic underestimation at high activity levels.

**Conclusions:**

This study demonstrates that highly accurate SPECT/CT-based ^161^Tb quantification is feasible, further cementing ^161^Tb as a viable theragnostic alternative. A MEGP collimator, a 75 keV window, and a CF derived from a homogeneous cylinder measurement appears preferable. The 49 keV window could be useful at late imaging time points, given its high sensitivity, if further optimized. Degradation from penetration and subsequent downscatter may be mitigated with a more refined reconstruction. Further investigations into dead-time effects are encouraged.

## Background

Although many nuclides have been proposed for targeted radionuclide therapy over the years, only a few have reached clinical success. In the past decade, radiopharmaceuticals labelled with lutetium-177 (^177^Lu) has become an established option for the treatment of neuroendocrine tumors (NETs) and metastatic castration-resistant prostate cancer (mCRPC). The success of ^177^Lu can be attributed to many factors, such as the feasibility of wide-scale production, favorable radiochemical properties, and, perhaps most importantly, attractive decay characteristics. Such characteristics include a suitable half-life (*T*_1/2_ = 6.65 d) and β^−^-particle energy (*E*_*av*_ = 134 keV), as well as γ-emissions compatible with single-photon emission computed tomography (SPECT) imaging (e.g., photon energy *hv* = 208 keV, emission probability 11%).

Recently, the β^−^-emitter terbium-161 (^161^Tb) has gained recognition as a viable alternative to ^177^Lu for the treatment of mCRPC and NETs. The two nuclides share many similarities; firstly, they both belong to the lanthanoid series, making their chemical properties nearly identical. This allows for effective radiolabeling using already established techniques [1]. Furthermore, ^161^Tb can be produced in the quantities and with the quality required for clinical use [1, 2].

In terms of decay properties, ^161^Tb exhibits a half-life (*T*_1/2_ = 6.95 d) [3] and an average β^−^ particle energy (*E*_*av*_ = 154 keV) similar to that of ^177^Lu, see Table 1. However, ^161^Tb also emits a significant proportion of low-energy, short-ranged conversion and Auger electrons (48.2 keV per decay, compared to 14.7 keV for ^177^Lu) [4]. This provides a unique dose deposition pattern, suitable for treating *both* larger tumors, as well as smaller metastases or even individual cancer cells [5–7]. Clinically, these additional short-range electrons are expected to enhance therapeutic efficacy, particularly for micrometastatic disease, making ^161^Tb an attractive alternative to ^177^Lu in both NET and mCRPC treatment.

In the context of NETs, ^161^Tb-labelled radiopharmaceuticals have shown promise; first-in-human studies with [^161^Tb]Tb-DOTATOC revealed a biodistribution matching that of ^177^Lu-labelled counterparts, with no reported adverse effects [8]. More recently, interest has been sparked in ^161^Tb-labelled somatostatin antagonists, such as DOTA-LM3, as opposed to the agonists currently used with ^177^Lu (e.g., DOTATATE and DOTATOC). Preclinically, both [^161^Tb]Tb-DOTATATE and [^161^Tb]Tb-DOTA-LM3 were found to produce a higher therapeutic efficacy, in vivo and in vitro, compared to their ^177^Lu-labelled counterparts [9]. A recent clinical case study reported promising results from a single 1 GBq infusion of [^161^Tb]Tb-DOTA-LM3 in a 78-year-old man (on-going trial; NCT05359146) [10].

The therapeutic potential of ^161^Tb has also been demonstrated for mCRPC. [^161^Tb]Tb-PSMA-617 was shown to outperform [^177^Lu]Lu-PSMA-617 in terms of both reduced in vitro cell viability and prolonged survival in mice [11]. Moreover, promising results have been observed clinically; a single [^161^Tb]Tb-PSMA-617 administration of 6.5 GBq produced notable partial remission for one 85-year-old man, pretreated with 8 cycles of [^177^Lu]Lu-PSMA-617 [12]. More clinical studies are currently underway (e.g., the VIOLET, REALITY, and PROGNOSTICS trials; NCT05521412, NCT04833517, and NCT06343038) [13].

While the therapeutic potential of the ^161^Tb particle emission pattern is clear, these decay characteristics present challenges for imaging. The significant emission of conversion and Auger electrons competes with the emission of characteristic X- and γ-rays, resulting in an intricate photon emission pattern, consisting of several intense, low-energy X-rays as well as numerous higher-energy, low-yield γ-emissions [14] (see Table 1). Such a complex emission profile can complicate SPECT imaging, particularly in terms of quantification.

The most abundant emissions, which have been suggested for imaging, are the 49 keV (17.1%) and 75 keV (10.3%) γ-photons. The first evaluation of clinical ^161^Tb SPECT/CT imaging, conducted by Marin et al. [15], reported that a 75 keV ± 10% photopeak window combined with a low-energy high-resolution collimator appeared preferable, producing images of comparable visual quality to those obtained with ^177^Lu. However, recovery coefficients obtained for different collimators did not align with visual assessments of spatial resolution, raising concerns about potential septal penetration. More recently, McIntosh et al. [16] confirmed that Marin’s proposed settings yielded good visual image quality, again comparable to ^177^Lu, but observed sensitivity variations across the activity range, suggesting dead-time issues. These effects were noted but not further investigated or explored, e.g., in terms of potential mitigation strategies.

Overall, these previous phantom-based evaluations have primarily focused on visual image quality metrics such as recovery, signal-to-noise ratio, and spatial resolution. However, the quantitative accuracy—particularly in relation to the choice of collimator, energy window, and reconstruction protocol—remains insufficiently characterized. Accurate quantification is a prerequisite for reliable image-based dosimetry, which is central to individualized treatment planning in targeted radionuclide therapy. To address this gap, the aim of the present study was to characterize the gamma-camera response in ^161^Tb SPECT/CT imaging and investigate the potential for accurate quantification, thereby assessing the feasibility of precise clinical image-based dosimetry.

**Table 1:** Decay properties of ^177^Lu and ^161^Tb, including decay mode, daughter nuclide, half-life (T_1/2_), mean emitted beta particle energy (Δ_β−_) per decay or the summed energy of conversion and Auger electrons (Δ_CE & Auger_) per decay [4], as well as γ and X-ray emissions and their corresponding emission yield [17, 18]

Isotope	^161^Tb	^177^Lu
Decay mode	β^−^ (100%)	β^−^ (100%)
$\:{T}_{1/2}$ (d)	6.95	6.65
Daughter	^161^Dy (Stable)	^177^Hf (Stable)
$\:{{\Delta\:}}_{{\beta\:}^{-}}$ (keV)	154	133
$\:{{\Delta\:}}_{\text{C}\text{E}\:\&\:\text{A}\text{u}\text{g}\text{e}\text{r}}$ (keV)	48.2	14.7
X-rays (keV) and yields	7.24 (22%) 45.2 (6.28%) 46.0 (11.2%) 52.2 (3.6%) 53.6 (0.94%)	8.93 (3.18%) 54.6 (1.59%) 55.8 (2.78%) 63.3 (0.917%) 65.1 (0.245%)
γ-emissions (keV) and yields	25.7 (23.2%) 28.7 (0.037%) 43.8 (0.06%) 48.9 (17.0%) 57.2 (1.78%) 59.2 (0.022%) 74.6 (10.2%) 77.4 (0.06%) 81.3 (0.0022%) 84.7 (0.0004%) 87.9 (0.183%) 101 (0.0001%) 103 (0.101%) 106 (0.078%) 113 (0.00011%) 132 (0.000102%) 138 (0.0008%) 213 (0.00004%) 239 (0.0023%) 286 (0.0143%) 292 (0.058%) 315 (0.0006%) 320 (0.0033%) 341 (0.0035%) 344 (0.0133%) 348 (0.0006%) 377 (0.00061%) 393 (0.0021%) 418 (0.0081%) 426 (0.0003%) 476 (0.0182%) 507 (0.0009%) 550 (0.036%)	71.6 (0.173%) 113 (6.20%) 137 (0.047%) 208 (10.38%) 250 (0.201%) 321 (0.216%)

## Methods

### Phantom preparations and activity measurements

**Fig. 1:**
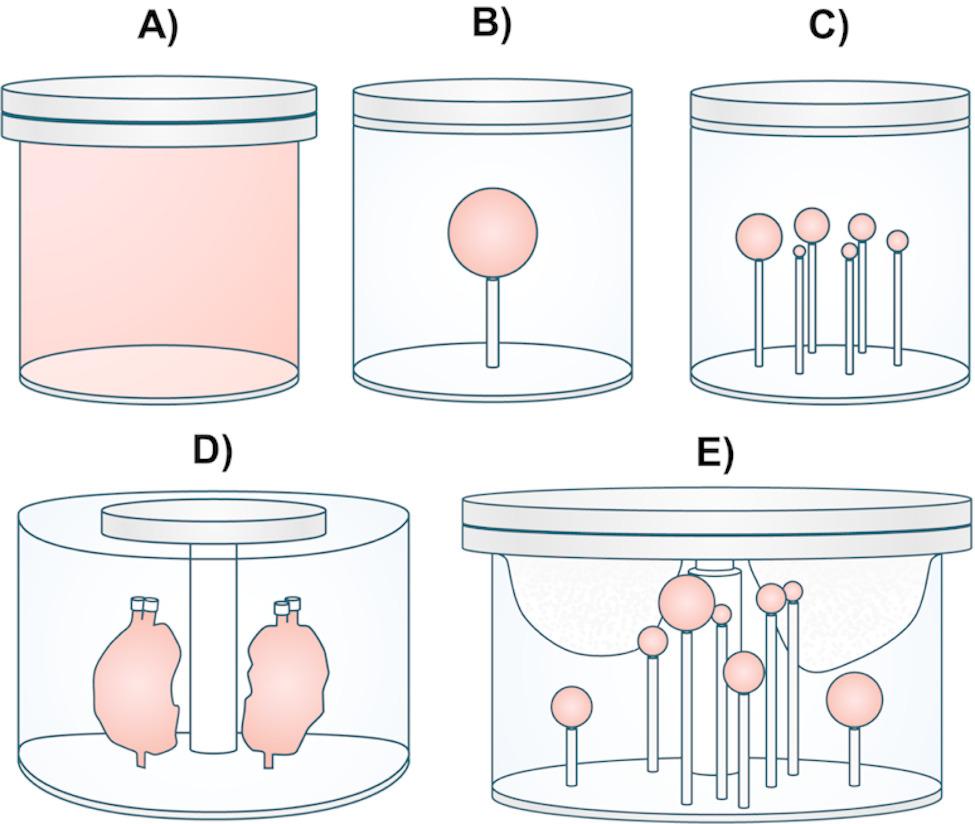
Schematic illustrations of the employed phantom geometries: **A**) Homogeneous Jaszczak SPECT Phantom™—used for SPECT sensitivity measurements and derivation of uniCFs; **B**) Flangeless Deluxe Jaszczak Phantom™ with 113-mL sphere insert—used for SPECT sensitivity measurements and derivation of sphCFs; **C**) Flangeless Deluxe Jaszczak Phantom™ with spherical inserts (V = 0.5–16 mL)—used for recovery and partial volume correction; **D**) NEMA Body Phantom™ with 3D-printed kidneys—used for visual image quality assessment; and **E**) Elliptical LungSpine Body Phantom™—used for quantitative assessments. Acquisition parameters, activity data, and additional measurement details are provided in Table 2

The phantom experiments were designed to evaluate different aspects of the image-based dosimetry workflow, from calibration factor derivation to recovery correction and quantification. Employed phantom geometries are illustrated in Fig. 1, with acquisition parameters, activity data, and measurement purposes summarized in Table 2.

**Table 2:** Summary of SPECT Phantom measurements, including acquisition times, activity data, and measurement purposes

Phantom	Time per projection (s) ELEGP/LEHR/MEGP	Activity concentration in insert (MBq/mL)	Activity concentration in background (kBq/mL)	Total activity (MBq)	Sphere-to-background ratio (SBR)	Purpose
Homogenous Jaszczak SPECT Phantom™	45/90/90	N/A	44.8	308	N/A	SPECT sensitivity/Calibration (denoted sphCF)
Flangeless Deluxe Jaszczak Phantom™ w/113-mL sphere	45/90/90	0.732	0	82.7	N/A	SPECT sensitivity/Calibration (denoted uniCF)
Flangeless Deluxe Jaszczak Phantom™ w/spherical inserts	45/90/90	0.855	0	26.9	N/A	Recovery and partial volume correction
NEMA Body Phantom™ w/3D-printed kindeys	30/60/60	0.923	0	245	N/A	Visual image quality assessments
Elliptical LungSpine Body Phantom™	30&60/60/60	1) 1.33 2) 1.21 3) 1.12	1) 0 2) 57.9 3) 103	1) 79.5 2) 459 3) 753	1) N/A 2) 20.8 3) 10.9	Quantitative assessments

Listed activity data corresponds to the initial imaging time-point. For sequential phantom measurements, acquisition times were adjusted to account for decay

For all experiments, non-carrier-added ^161^Tb was produced at the Paul Scherrer Institute (PSI), Villigen-PSI, Switzerland, and shipped to Sahlgrenska University Hospital, Gothenburg, Sweden. Activity measurements were performed using a VDC-505 Dose Calibrator (Veenstra Instruments), calibrated to ^161^Tb in a 10 mL glass vial (cross-calibrated to a PSI reference). Activity concentrations were determined using the subtraction method, i.e., by measuring the 10 mL reference vial before and after transfer. Insert volumes (and thus total activities, given the known concentration) were obtained by weighing each insert before and after filling on a precision scale (Sartorius AX423, accuracy = 1 mg). Pentetic acid (4 g/L, ≥ 98%) was added to all phantoms solutions to prevent activity from adhering to the inner walls.

### Imaging equipment and general acquisition parameters

Imaging was performed using a Discovery NM/CT 670 Pro system (GE Healthcare) with a 15.9 mm (5/8”) NaI(Tl) crystal. Three collimator options were employed: extended low-energy general-purpose (ELEGP), low-energy high-resolution (LEHR), and medium-energy general-purpose (MEGP).

The energy window sequence proposed by Marin et al. [15] was employed, consisting of two main energy windows over the 49 and 75 keV γ-peaks (EM_1_ = 40.7–62.9 keV and EM_2_ = 67.1–82.1 keV, respectively) and three adjacent scattering windows (SC_1_ = 38.2–40.6 keV, SC_2_ = 63.0–67.0 keV, and SC_3_ = 82.2–87.2 keV keV).

To ensure optimal quantitative conditions, extrinsic uniformity maps were acquired for each collimator using a ^161^Tb-filled flood source phantom (*A* ≈ 600 MBq, *V* = 6.5 L), as previously recommended [15]. Measurements were conducted in H-mode (i.e., with the detectors facing each other) at a 10 cm source-to-collimator distance, using a 256 × 256 matrix and 60,000 kcts per designated photopeak window (i.e., EM_1_ and EM_2_, separately).

### SPECT/CT acquisition parameters

All SPECT acquisitions were performed using a conventional step-and-shoot technique (360° angular range, 180°/detector) with 120 projections (3°/view), a 256 × 256 matrix (2.21 × 2.21 mm pixels), and automatic body contouring. The time per projection was chosen by virtue of the desired statistical accuracy. When measuring the same phantom sequentially, acquisition times were adjusted to account for physical decay. Due to the higher sensitivity, measurements with ELEGP were performed with a reduced acquisition time compared to LEHR and MEGP, giving similar count statistics for all three collimators.

Moreover, CT images were acquired using a clinical low-dose protocol, with a tube voltage of 120 kV, smart tube current of 30–80 mA, matrix size of 512 × 512 (0.98 × 0.98 mm pixels), and slice thickness of 2.00–1.25 mm (smaller for easier delineation in phantoms containing inserts).

SPECT reconstructions were performed using a clinical ordered-subset expectation-maximization (OSEM) algorithm (Xeleris 4.0, GE), including an attenuation correction, a triple energy window (TEW) correction, and resolution recovery, using 10 subsets and 2–20 iterations (every other iteration saved).

### Calibration factor evaluation

To evaluate the SPECT system sensitivity, and derive calibration factors (CFs) for quantification, two separate phantom setups were used: (1) a cylindrical Jaszczak SPECT Phantom™ (*V* = 6.89 L) with uniform activity (denoted uniCF) and (2) a large, activity-filled sphere (*V* = 113 mL) in a Flangeless Deluxe Jaszczak Phantom™ with no background activity (denoted sphCF; details in Table 2). The sphere setup has the advantages of requiring less activity and being easier to handle, compared to the homogeneous phantom. For the two setups, CFs were determined as follows:

1 $$\:CF=\frac{\dot{C}}{{A}_{Ref}}\:$$

where $\:\dot{C}$ is the image count rate (cps) within a delineated volume-of-interest (VOI), and $\:{A}_{Ref}$ (MBq) is the true activity in that same region (i.e., activity concentration as measured by the dose calibrator multiplied by VOI size, or total activity for VOIs exceeding the phantom).

**Fig. 2:**
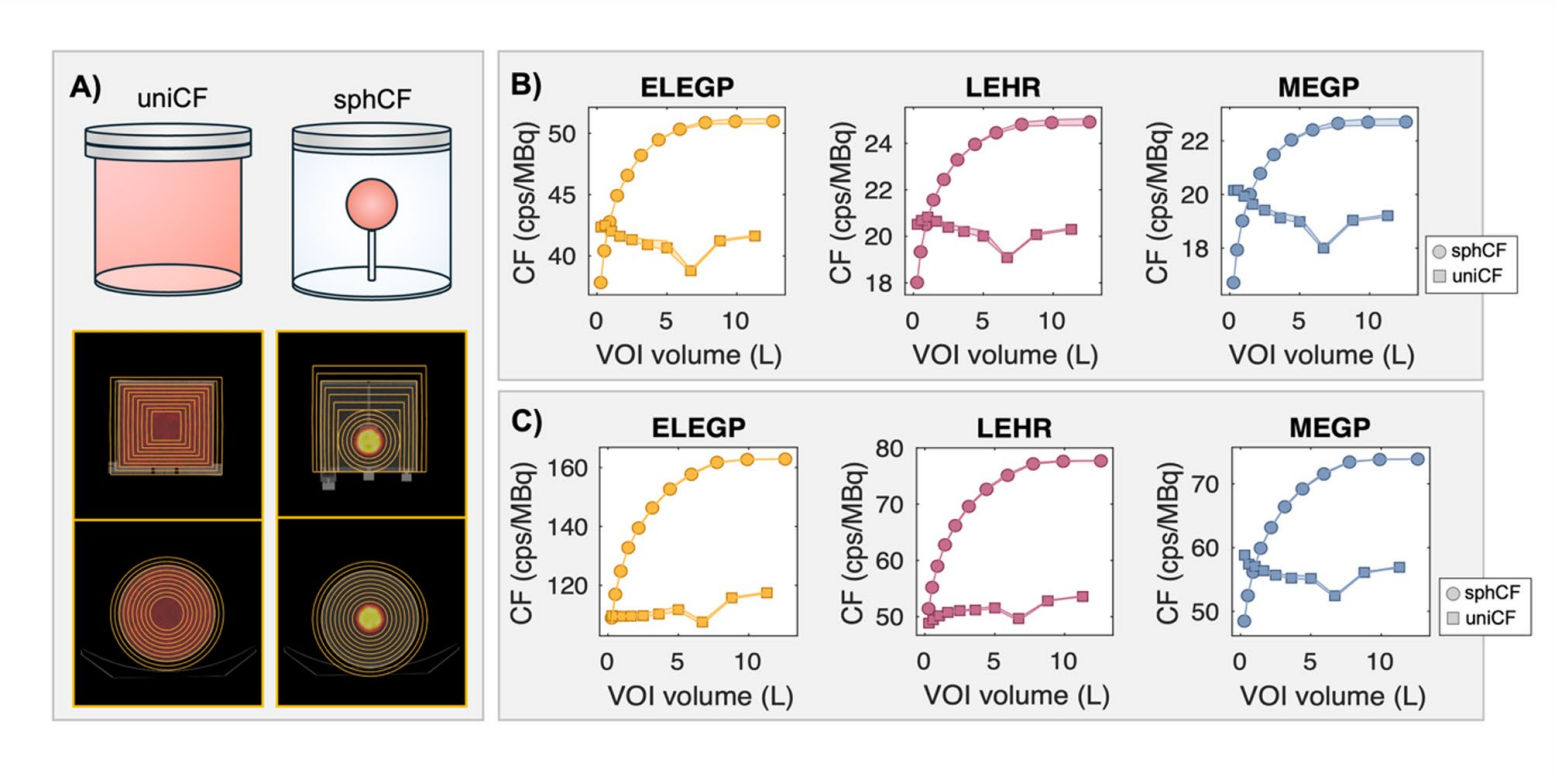
**A**) Experimental setup, including schematic illustrations of the Homogeneous Jaszczak SPECT Phantom™ (used for determining uniCFs) and the Flangeless Deluxe Jaszczak Phantom™ with the 113-mL sphere insert (used for determining sphCFs), along with transaxial and coronal SPECT/CT slices showing the employed VOIs. **B**–**C**) Resulting CFs as a function of VOI volume for the 75 keV (**B**) and 49 keV (**C**) windows for all examined collimators. Square markers denote uniCFs, sphere markers denote sphCFs, and shaded areas indicate variations due to the number of iterations

To examine the robustness of the two CF setups, different VOI geometries were employed in Eq. (1). For the homogenous phantom, a range of uniCFs was obtained using cylindrical VOIs of increasing size (*n* = 10, *V* = 0.297–11.3 L) positioned at the center of the phantom, with the two largest VOIs exceeding the phantoms physical dimensions of the phantom (Fig. 2A).

A similar approach was used to obtain a range of sphCFs, starting with spherical VOIs centered on the insert and incrementally increasing in radius (*n* = 4, *V* = 0.268–1.44 L). Once the spherical VOI reached the phantom’s outer edge, cylindrical VOIs were used (*n* = 7, *V* = 2.18–12.6 L) (Fig. 2A).

### Recovery measurements

#### Kidney recovery coefficients

For visual image quality assessment, two 3D-printed kidney inserts—one with a cortex compartment (*V* = 107 mL) and one with a combined cortex and medulla compartment (*V* = 158 mL)—were imaged in a NEMA Body Phantom™ along with a spinal insert and a cold background (details in Table 2). The RCs were determined as the fraction between measured and true activity as follows:

2 $$\:RC=\frac{A}{{A}_{Ref}}=\frac{\dot{C}\times\:\frac{1}{CF}}{{A}_{Ref}}\:$$

where $\:\dot{C}$ is the count rate within a VOI matching the physical dimensions of the insert, $\:{A}_{Ref}$ is the inserted activity as measured by the dose calibrator. Different CFs were evaluated. Ultimately, the sphCF with a 3 cm VOI margin was chosen, as this produced RCs most consistent with the observed spatial resolution, regardless of collimator selection or energy window setting.

#### Sphere-based recovery coefficients

To determine RCs for partial volume correction (PVC), a Flangeless Deluxe Jaszczak Phantom™ with six spherical inserts (*V* = 0.5, 1, 2, 4, 8, and 16 mL) was used with no added background activity (details in Table 2). The RCs were determined with Eq. (2).

### Quantitative evaluation

To evaluate quantifiability in a more complex geometry, mimicking a more patient-like scenario, measurements were performed using an Elliptical LungSpine Body Phantom™ (hollow cylinder, *V* ≈ 9.4 L) with eight spherical inserts (*V* = 2, 4, 8, and 16 mL, two of each). The spheres were mounted at three different heights (3, 6, and 9 cm), at various lateral positions, as displayed in Fig. 2. The phantom also contained two lung inserts filled with Styrofoam beads and water, as well as a solid Teflon spinal rod insert. The phantom was imaged at three different sphere-to-background ratios (SBRs): infinity (cold background), 20:1 (459 MBq in background) and 10:1 (753 MBq in background). Due to the considerably higher sensitivity of ELEGP compared to LEHR and MEGP, duplicate measurements were conducted for this collimator to assess potential noise-related errors (see Table 2).

The relative quantification error (RQE) was calculated for each insert as follows:

3 $$\:RQE=\frac{A-{A}_{Ref}}{{A}_{Ref}}=\frac{\dot{C}\times\:\frac{1}{CF}\times\:\frac{1}{RC}}{{A}_{Ref}}-1\:$$

where RC represents the sphere-based RCs from Eq. (2). All other notations remain consistent with Eq. (2).

## Results

### Calibration factor evaluation

The effect of energy window, collimator, and number of OSEM updates on SPECT volume sensitivity was evaluated from sphCFs and uniCFs (minimum, maximum, and median; Table 3) and their dependence on VOI size (Fig. 2B). Generally, only minor variations were observed in relation to the number of OSEM updates. On average, CFs were approximately 3-fold higher for the 49 keV window compared to the 75 keV window. Additionally, the CFs were roughly 2-fold higher for ELEGP compared to MEGP and LEHR. For the sphCFs, a distinct increase with VOI size was evident, consistent across collimators (35–38% for 75 keV, 49–52% for 49 keV). The uniCFs showed no apparent volume-dependent trend, and a lower dependence of VOI size generally. That said, a slight decrease in uniCFs (1.8–7.2% compared with their respective medians; Fig. 2B) was observed when the VOI matched the exact physical dimensions of the phantom activity.

**Table 3:** Median SPECT CFs (cps/MBq) and ranges for different VOI sizes, from the homogeneous cylinder (uniCF) and sphere-based (sphCF) phantoms, for each collimator–energy window combination

Energy window	Collimator	uniCF (cps/MBq)	Range (min–max)	sphCF (cps/MBq)	Range (min–max)
75 keV	ELEGP	41.6	(38.8–42.5)	48.8	(37.8–51.7)
	LEHR	20.4	(19.1–21.0)	23.6	(18.0–25.7)
	MEGP	19.4	(18.0–20.2)	21.8	(16.7–23.0)
49 keV	ELEGP	110	(108–120)	150	(109–165)
	LEHR	51.1	(48.9–55.3)	71.2	(51.4–79.1)
	MEGP	56.4	(52.4–58.8)	67.8	(48.5–74.8)

### Recovery measurements and visual image quality assessments

**Fig. 3:**
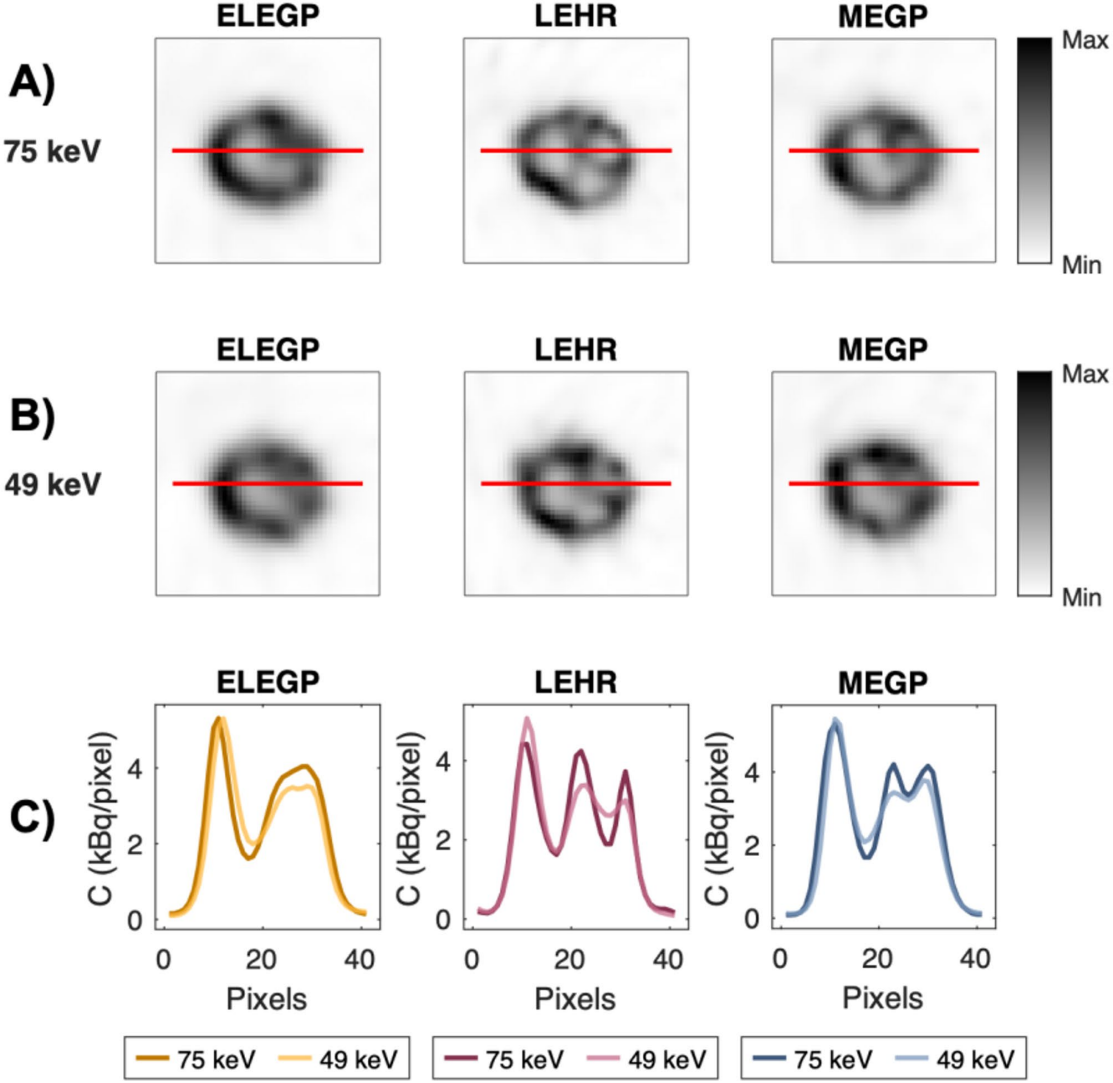
Axial slices of one of the 3D-printed kidney inserts (cortex compartment only, *V* = 107 mL) reconstructed with 4i10s, at **A**) 75 keV and **B**) 49 keV, with extracted lineprofiles in **C**)

**Fig. 4:**
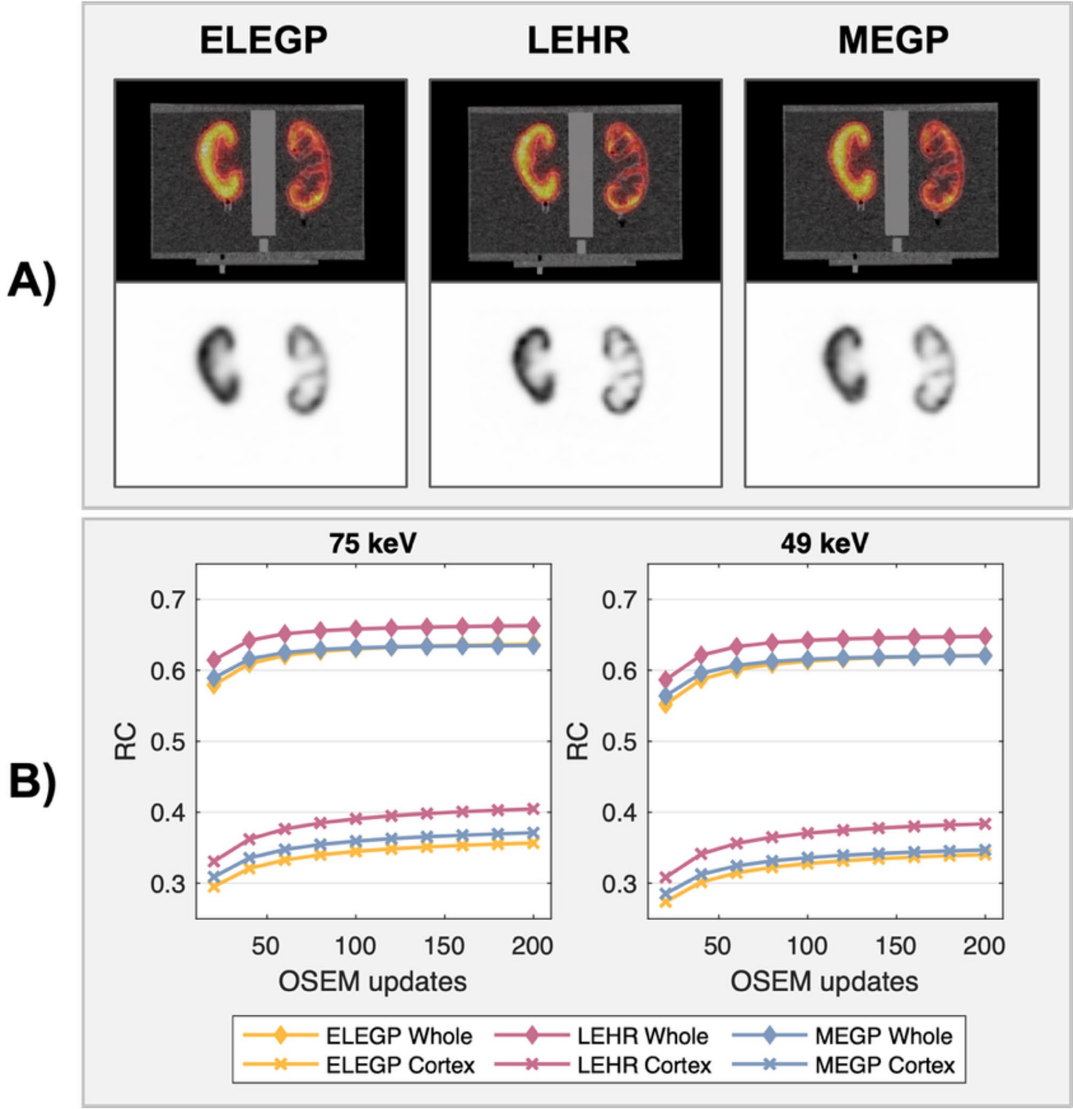
**A**) Coronal slices of the NEMA Body phantom with the 3D-printed kidney inserts for the ELEGP, LEHR, and MEGP collimators for the 75 keV window, reconstructed with 4i10s. **B**) RCs as a function of the number of OSEM updates for the inserts at 75 and 49 keV, respectively

In Fig. 3, axial slices of the kidney cortex insert are shown for the different collimators at 49 and 75 keV, respectively. Visually, the LEHR collimator demonstrated higher spatial resolution compared to ELEGP and MEGP. Extracted line profiles support this observation (Fig. 3C). Additionally, improved contrast and resolution was seen at 75 keV across all collimators, compared to 49 keV. Derived RCs were consistent with these findings: highest for LEHR, followed by MEGP and ELEGP, and lower at 49 keV compared to 75 keV for all collimators (Fig. 4).

**Fig. 5:**
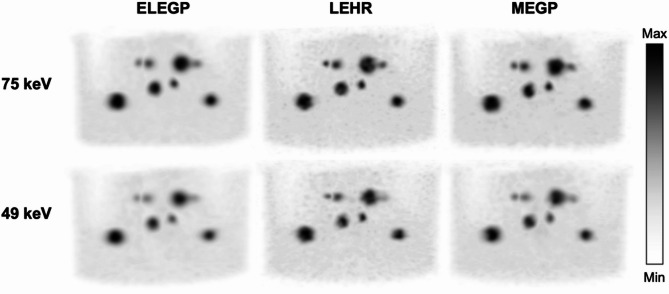
Maximum intensity projections (MIPs) of the LungSpine phantom with SBR = 10:1 for all three collimators at 49 and 75 keV, respectively, reconstructed with 4i10s

**Fig. 6:**
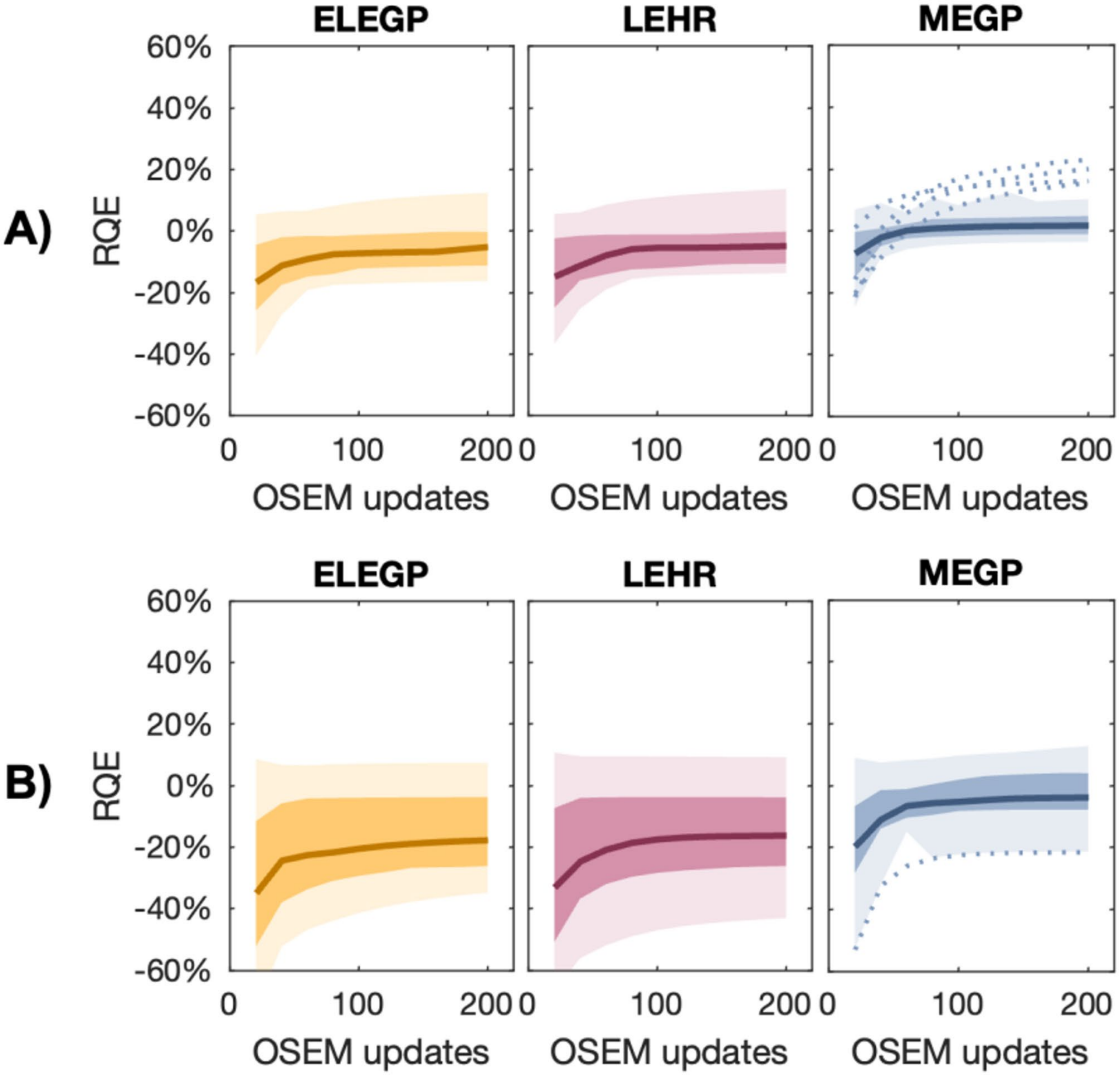
The relative quantification error (RQE) as a function of the number of OSEM updates for the three different collimators, all LungSpine measurements combined (*n* = 24, 8 spheres × 3 SBRs). Panel **A**) RQEs at 75 keV, and **B**) RQEs at 49 keV. Dark-shaded areas indicate the interquartile range (IQR), and the light-shaded areas mark the 1.5 IQR. Series containing outliers are visualized by dotted lines

In Fig. 5, SPECT images of the LungSpine phantom (displayed as maximum intensity projections) are shown for each collimator for both energy windows. Figure 6 shows the RQE as a function of OSEM updates, all LungSpine measurements combined (*n* = 24, 8 spheres × 3 SBRs). Similar trends were observed for both energy windows, though errors were generally larger at 49 keV. No significant difference was found between the ELEGP measurements with 30 s and 60 s per projection; therefore, only results from the longer acquisition are presented.

All individual RQE estimates, along with the median RQE (mRQE), the interquartile range (IQR) and maximum and minimum errors (as visualized in Fig. 6) at 20i10s updates, are shown in Table 4. The smallest mRQE, as well as the smallest IQR and total range, was achieved with MEGP for the 75 keV window (mRQE = 1.9%, IQR = [0.92%, 4.8%]). A two-sided Wilcoxon signed-rank test revealed statistically significant differences between MEGP and LEHR as well as between MEGP and ELEGP (*p* ≤ 0.001) for both energy windows.

**Table 4.**
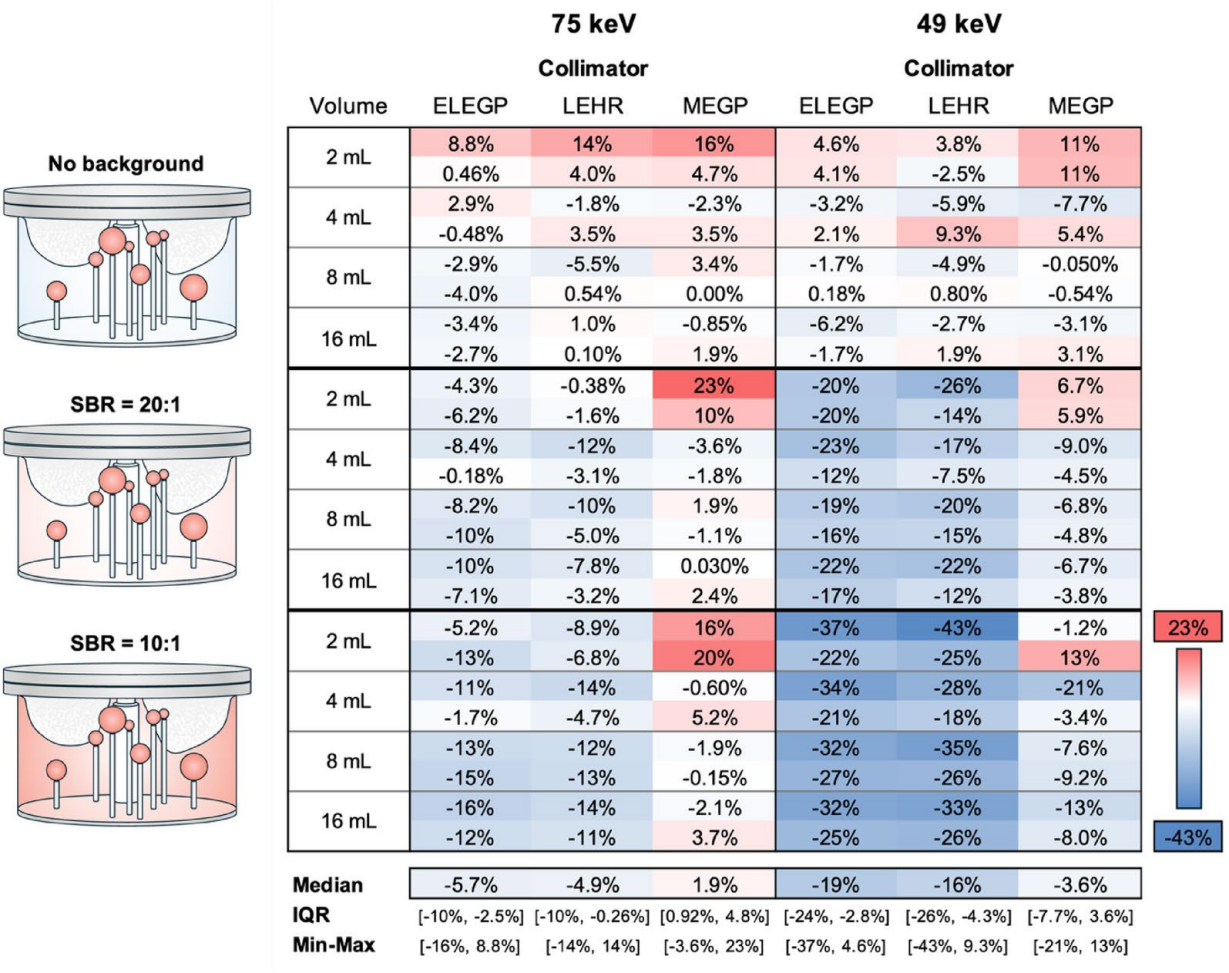
Individual RQEs for all inserts at 20i10s at 75 and 49 keV, respectively

The bottom row shows the median RQE, the interquartile range (IQR) and the maximum and minimum errors for each collimator and window combination, as displayed in Fig. 6

**Fig. 7:**
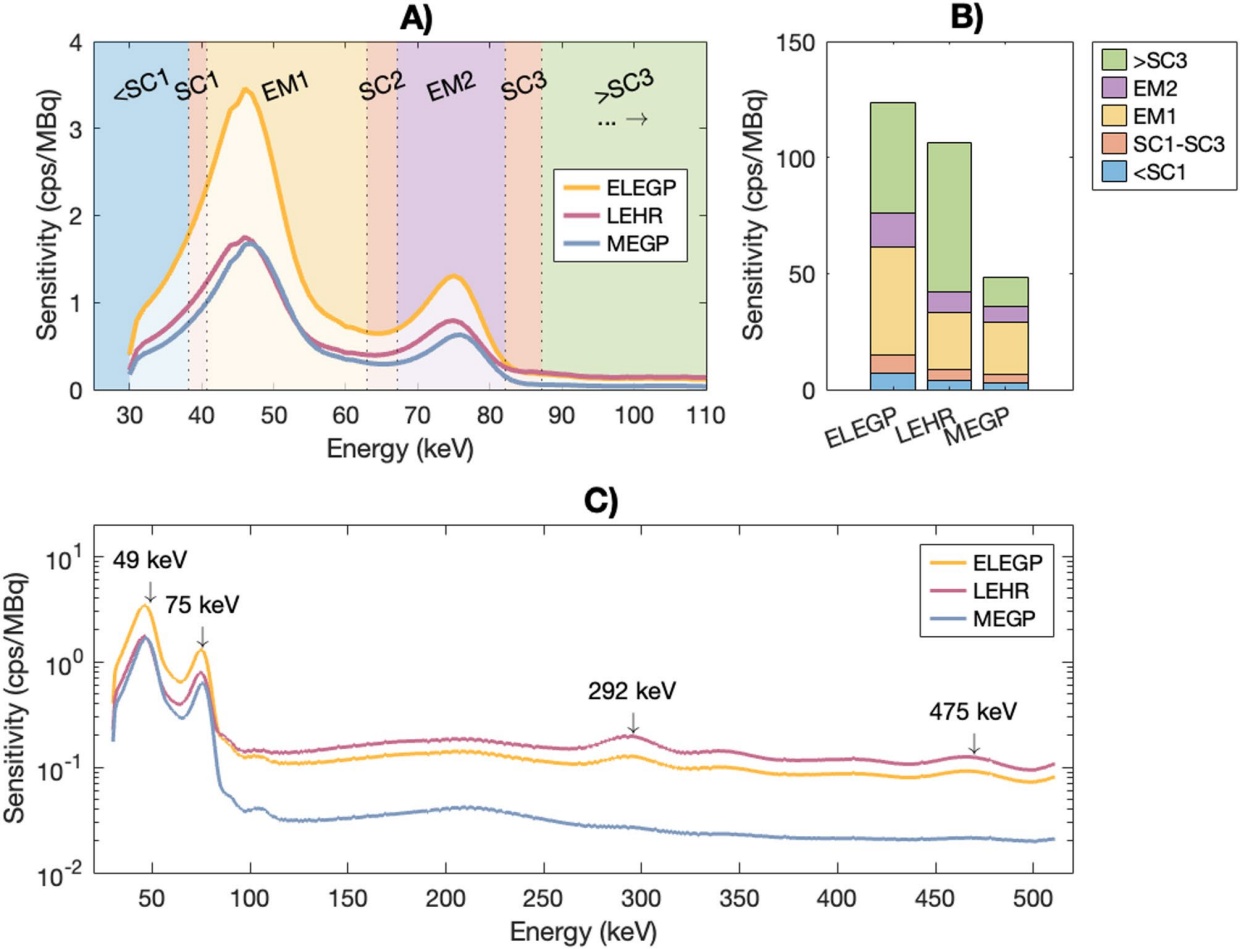
Energy spectra from the LungSpine measurements with SBR = 10:1. In **A**), spectra for each collimator are shown along with the employed energy window settings. The bar plots in **B**) show the count rate recorded across different energy regions. In **C**), the full spectra (32–511 keV) are displayed in logarithmic scale, with arrows indicating notable peaks

Figure 7 shows listmode spectra from the LungSpine measurement with a SBR of 10:1. For LEHR and ELEGP, peaks appeared at 292 and 475 keV (emission probabilities of 0.058% and 0.018%). These were suppressed with MEGP (Fig. 7C). Additionally, 60% of recorded counts for LEHR were above the upper scatter window (> SC3), compared to 38% for ELEGP and 26% for MEGP (Fig. 7B).

## Discussion

In this study, we have demonstrated that ^161^Tb SPECT/CT imaging with good quantitative accuracy is feasible. However, the findings highlight several crucial aspects that require attention.

### Calibration

When examining the derived SPECT CFs (Fig. 2), it becomes evident that both measurement and VOI geometry have a significant effect on the outcome. For the cylinder phantom with uniform activity, the chosen VOI had little impact on the uniCFs, showing no volume-related trends and a mean absolute deviation below 3% across all collimators and energy windows.

In contrast, the sphCFs (derived from the sphere-based setup) increased considerably with increasing VOI size, which can be attributed to insufficient compensation of scatter. The inclusion of scattered counts causes the sphCFs to increase, eventually reaching near-constant values when the VOI reaches the phantom’s outer edge; a trend that renders this otherwise simple method for determining CF unsuitable in practice (i.e., unless scatter is perfectly compensated for).

Nevertheless, the CF analysis reveals some interesting findings. Firstly, the low-energy collimators were more affected by scattered photons than the medium-energy collimator. For the 75 keV window, the sphCF/uniCF ratio for the largest VOI was approximately 1.23 for the low-energy collimators, compared to 1.17 with MEGP, indicating a higher amount of scatter. This aligns with the acquired listmode spectra, which show nearly ten times higher penetration (in terms of count rate above 100 keV) for low-energy collimators compared to MEGP (Fig. 7), resulting in scatter contaminations within the photopeaks. This effect is even more pronounced in the 49 keV window, where the sphCF/uniCF ratio rises to about 1.46 and 1.52 for the ELEGP and LEHR collimators, and 1.31 for MEGP collimator.

In more complex measurement scenarios, as when determining the biodistribution of radiopharmaceuticals in patients, minimizing the influence of scattered high-energy photons is crucial for obtaining good quantitative accuracy. This analysis suggests that the MEGP collimator, combined with a 75 keV window and a uniCF would be a preferable choice over other alternatives.

### Kidney measurements

In the kidney phantom, differences in visual image quality between collimators are especially noticeable; as expected, the LEHR collimator offers significantly better spatial resolution than the MEGP and ELEGP collimators, as demonstrated by higher RCs (Fig. 4) and sharper, more defined line profiles (see Fig. 3). Furthermore, better contrast and resolution can be observed for 75 keV compared to 49 keV for all collimators.

Applying different CFs to derive the kidney RCs in Eq. (2) also provides insight into the adequacy of the calibration. For ELEGP and LEHR, using uniCFs results in higher RCs at 49 keV than at 75 keV, despite the lower resolution (Suppl. Figure 1). In contrast, applying sphCFs yields RCs that align better with visual observations and line profiles. This discrepancy once again suggests insufficient compensation of scatter. For this scenario specifically, the sphere-based calibration geometry appears to better approximate the scatter properties of the kidney phantom, thus producing more reasonable RCs. Notably, this discrepancy does not occur with the MEGP collimator, where RCs remain similar regardless of the chosen calibration geometry. This is likely due to its more effective elimination of penetration and subsequent downscatter, once again underscoring the superiority of medium-energy collimation.

### Quantitative analysis

Moving to the quantitative analysis, the anthropomorphic phantom study revealed better accuracy (i.e., RQEs closer to zeros) for MEGP, compared to both low-energy collimators. Similar trends for both energy windows; however, the errors were generally larger for the 49 keV window (Fig. 6).

It should be noted that the choice of CF does not affect the RQEs, as a PVC is applied. The RCs used for the PVC were determined using the same CF as in the LungSpine quantifications. Thus, the CF in Eq. (3) cancels out. Consequently, the RQE reflects the difference in RC between the calibration and evaluation measurements. From this perspective, the MEGP collimator provides more consistent RCs, regardless of measurement geometry or activity background level, offering better conditions for accurate quantification.

Despite the generally favorable results, a few outliers appear for the MEGP collimator (dotted lines in Fig. 6, with corresponding estimates in Table 3). In all cases, these outliers (i.e., overestimations) correspond to measurements of the smallest spheres (V = 2 mL). This systematic overestimation (Table 3) is likely due to an underestimation of the 2 mL RCs in the Jaszczak measurements (Suppl. Fig. 2). This highlights a drawback of MEGP collimation: reduced spatial resolution, complicating quantification for smaller lesions.

Looking at the measurements with different background activity levels individually, a systematic underestimation is observed as background activity increases (Table 3)—a trend consistent with dead-time effects. The underestimations were more pronounced for the low-energy collimators compared to for MEGP, which is reasonable given considerably higher whole-spectrum count rate observed for LEHR and ELEGP (caused by septal penetration; see Fig. 7). In addition, the underestimations were more severe for the 49 keV window (Table 3), which is consistent with pile-up effects. This supports that the reduced accuracy observed for the low-energy collimators is indeed due to dead-time losses.

Generally, dead time appears to require careful consideration in ^161^Tb SPECT imaging. The combination of intense low-energy X-rays and potential septal penetration from high-energy photons results in a high whole-spectrum count rate compared to established radionuclides such as ^177^Lu. As a result, dead-time effects are more likely to occur at lower activities. In a study by McIntosh et al. (2024), dead effects were observed for activities above 2 GBq in measurements of ^161^Tb on a Siemens Symbia Intevo Bold SPECT/CT scanner [16], whereas our findings suggest that such effects may be prominent at activities as low as ~ 450 MBq (i.e., as seen for SBR = 20:1, Table 3). Further investigations are therefore strongly encouraged.

### Limitations

In this study, all imaging was performed on a GE Discovery NM/CT 670 Pro system with a 5/8” NaI(Tl) crystal, with the imaging protocol proposed by Marin et al. (2020). The energy window settings in this protocol have not been evaluated nor optimized. While the 75 keV ± 10% adheres to general recommendations, the settings for the 49 keV window are evidently inappropriate. The window is shifted towards higher energies, resulting in a significant proportion of the recorded signal consisting of down-scattered counts from the 75 keV, see Fig. 7.

To fairly evaluate the quantitative potential of the 49 keV window, alterations to the energy window settings are necessary. It is important to note that the 49 keV window includes multiple other X-ray peaks, e.g., 45.2 keV (6.3%), 46.0 keV (11.3%), and 52.2 keV (3.6%), which should be considered when selecting the window width. However, as these energies fall outside the usual operating range of most gamma-camera systems, validation by vendors is potentially necessary.

However, despite the suboptimal window settings, the 49 keV window does show promise in terms of quantification. Although the SNR is lower for this energy window compared to the 75 keV [15], the higher sensitivity—3-fold greater than the 75 keV window—could make it advantageous for quantification at late time points, especially for low-uptake areas such as the bone marrow. In the present stucy, decent accuracy was obtained for the MEGP collimator (mRQE = −3.6%, IQR = [−7.7%, 3.6%]; Table 3). However, degradation from increased scattering was observed, which would have to be mitigated with a more refined reconstruction.

Concerning other limitations of this study, the DICOM nuclide chosen for the acquisition protocol was ^201^Tl (*hv* = 71 keV), as the system is not validated for ^161^Tb. It should also be noted that the employed crystal thickness of 5/8” may not be optimal for ^161^Tb imaging due to its low-energy emissions. A thinner crystal could improve resolution and potentially reduce contributions from high-energy emissions.

Reconstructions were performed within the Xeleris 4.0 workstation, using the Volumetrix application. No efforts were made to optimize the window-based scatter correction in terms of scaling or altering the window widths. Instead, default scatter correction settings were employed. Optimizing the window settings could increase accuracy for both energy window settings, especially for the low-energy collimators, for which the projection data is more degraded from downscatter.

### Other considerations

As for any radionuclide, accurate quantification starts with proper calibration of the dose calibrator. This is particularly important for low-energy photon emitters like ^161^Tb, where photon attenuation is more prominent. Juget et al. observed significant response differences in dose calibrator measurements of ^161^Tb in different containers, with Eppendorf tubes yielding higher responses than sterile glass or GMP vials (8.5% and 14%, respectively). Even the same type of penicillin vial showed up to a 4.5% signal variation due to glass irregularities [19]. Thus, geometry must be carefully considered in ^161^Tb dose calibrator measurements. Additionally, impurities like ^160^Tb and its high-energy emissions should be considered, as contributions from this nuclide could seriously degrade image quality. Its long half-life also necessitates careful management in clinical radiation protection and waste disposal [2].

## Conclusions

This study demonstrates that achieving good quantitative SPECT/CT accuracy with ^161^Tb is feasible. The 75 keV ± 10% window provided the best results, with MEGP outperforming the LEHR and ELEGP collimators. The reduced accuracy observed with low-energy collimation can partially attributed to degradation from penetration and subsequent downscatter. This being said, the LEHR collimator yielded the best visual image quality in terms of spatial resolution.

Similar trends were observed for the 49 keV window, compared to the 75 keV window, but errors were generally larger, likely due to increased scattering and pulse pile-up. With further optimization, the 49 keV window could prove valuable for quantification at late time points, given its significantly higher sensitivity.

A systematic underestimation of activity was noted at higher background activity levels, likely due to dead time effects. This may result from intense X-ray emissions combined with penetration and scatter contributions—effects that were more pronounced with LEHR and ELEGP collimation, due to the additional contributions from penetration. Further investigations into dead-time effects are recommended.

## Supplementary Information

Supplementary Material 1.

## Data Availability

The datasets used and/or analyzed during the current study are available from the corresponding author on reasonable request.

## Abbreviations

^161^Tb: Terbium-161
^177^Lu: Lutetium-177
CF: Calibration factor
ELEGP: Extended low-energy general-purpose
EM: Main energy window
IQR: Interquartile range
LEHR: Low-energy high-resolution
mCRPC: Metastatic castration-resistant prostate cancer
MEGP: Medium-energy general-purpose
mRQE: Median relative quantification error
NETs: Neuroendocrine tumors
PVC: Partial volume correction
RC: Recovery coefficient
RQE: Relative quantification error
SBR: Sphere-to-background ratio
SC: Scattering window
SPECT: Single-photon emission computed tomography
sphCF: Sphere-based calibration factor
TEW: Triple energy window
uniCF: Uniform calibration factor
VOI: Volume-of-interest
