# Supplementary material for: Quantitative terbium-161 SPECT/CT imaging: demonstrating the feasibility of image-based dosimetry and highlighting pitfalls
Source: EJNMMI Res. 2025 Oct 13;15:130. doi: 10.1186/s13550-025-01326-3 (PMC12518732; doi:10.1186/s13550-025-01326-3)
Supplement: Supplementary file 1 — Supplementary Material 1. [file 13550_2025_1326_MOESM1_ESM.docx]

**Supplements**

Figure 1: Kidney recovery coefficients (cortex compartment only; *V* = 107 mL) determined with the sphCF and uniCF for the 75 and 49 keV window, respectively.


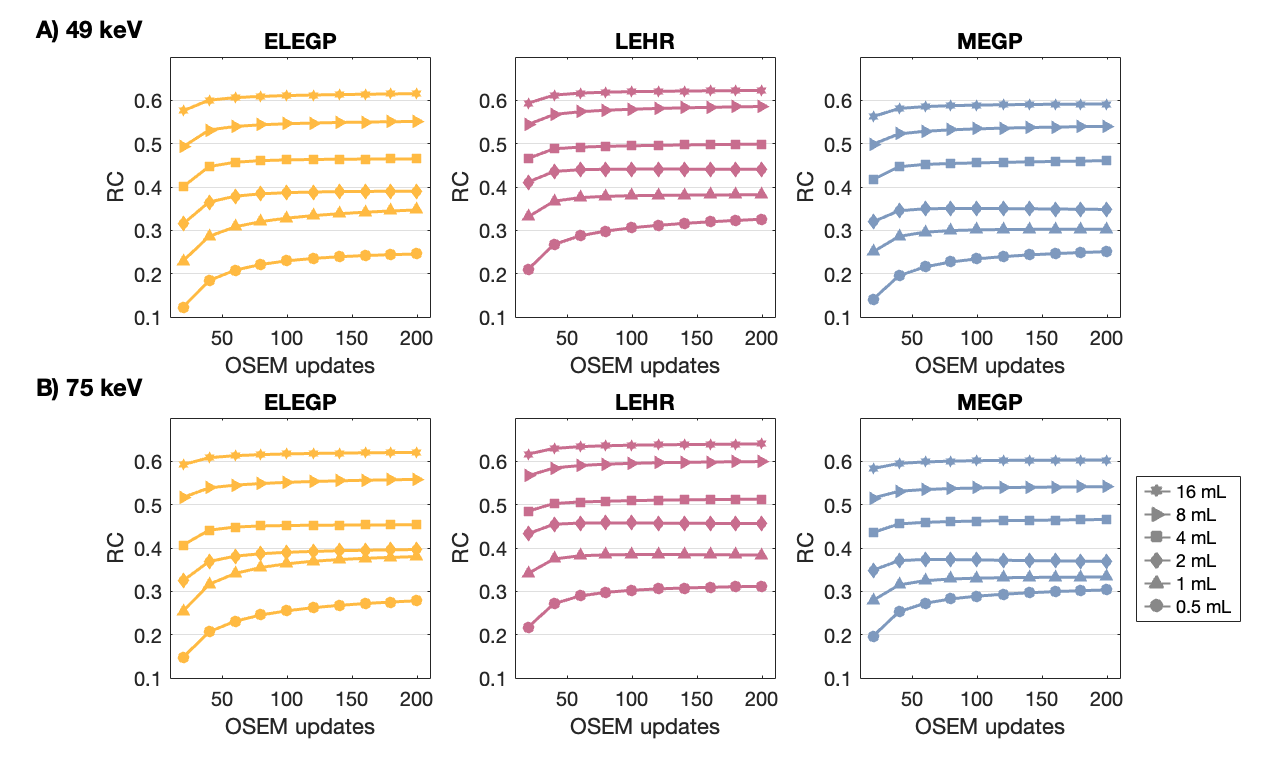


Figure 2: Jaszczak RCs used for the partial volume correction for the 75 and 49 keV window, respectively, determined using the sphCF.
